# Enhancing the production of PHA in *Scenedesmus* sp. by the addition of green synthesized nitrogen, phosphorus, and nitrogen–phosphorus-doped carbon dots

**DOI:** 10.1186/s13068-024-02522-4

**Published:** 2024-06-04

**Authors:** Pablo Alfredo Sánchez-Pineda, Itzel Y. López-Pacheco, Angel M. Villalba-Rodríguez, José Alfonso Godínez-Alemán, Reyna Berenice González-González, Roberto Parra-Saldívar, Hafiz M. N. Iqbal

**Affiliations:** 1 Tecnologico de Monterrey, School of Engineering and Sciences, 64849 Monterrey, Mexico; 2 Tecnologico de Monterrey, Institute of Advanced Materials for Sustainable Manufacturing, 64849 Monterrey, Mexico

**Keywords:** Nanomaterials, Microalgae, Polyhydroxyalkanoates, Bioplastic, Nanotechnology, *Scenedesmus* sp.

## Abstract

Plastic consumption has increased globally, and environmental issues associated with it have only gotten more severe; as a result, the search for environmentally friendly alternatives has intensified. Polyhydroxyalkanoates (PHA), as biopolymers produced by microalgae, might be an excellent option; however, large-scale production is a relevant barrier that hinders their application. Recently, innovative materials such as carbon dots (CDs) have been explored to enhance PHA production sustainably. This study added green synthesized multi-doped CDs to *Scenedesmus* sp. microalgae cultures to improve PHA production. Prickly pear was selected as the carbon precursor for the hydrothermally synthesized CDs doped with nitrogen, phosphorous, and nitrogen–phosphorous elements. CDs were characterized by different techniques, such as FTIR, SEM, ζ potential, UV–Vis, and XRD. They exhibited a semi-crystalline structure with high concentrations of carboxylic groups on their surface and other elements, such as copper and phosphorus. A medium without nitrogen and phosphorous was used as a control to compare CDs-enriched mediums. Cultures regarding biomass growth, carbohydrates, lipids, proteins, and PHA content were analyzed. The obtained results demonstrated that CDs-enriched cultures produced higher content of biomass and PHA; CDs-enriched cultures presented an increase of 26.9% in PHA concentration and an increase of 32% in terms of cell growth compared to the standard cultures.

## Introduction

Plastic production and plastic waste have increased dramatically in recent years. Global plastic production has doubled in the last two decades, and the current amount is estimated to triple by 2060 [1]. Furthermore, only about 9% of it is successfully recycled; most plastic waste (79%) ends up in landfills or the environment, while approximately 12% is incinerated [2]. Moreover, the improper disposal of plastics into the environment negatively impacts the entire ecosystem [3]. Plastic can persist for long periods in the environment, breaking down and transforming into microplastics and nano-plastics, which are harder to remove from nature [4, 5]. Micro- and nano-plastics have been detected in a large diversity of species of animals, including marine organisms; thus, micro- and nano-plastics have entered the food chain [6, 7].

Bio-based plastics or bioplastics are at the forefront as a solution for the plastic waste problem; they are biodegradable materials produced from natural or renewable sources. Bioplastics are excellent substitutes for petroleum-based plastics because of their eco-friendliness, sustainability, and biodegradability. Various sources have been explored for synthesizing bioplastics, including polysaccharides [8–10], proteins [11, 12] and microorganisms [13–15]. Recently, microorganisms have attracted attention due to their rapid growth, high biomass accumulation, minimal nutrient requirements, and natural abundance [16]. Within microorganisms, microalgae distinguish themselves not only for their bioplastic production potential [17] but also for water treatment applications [18] and the production of biofertilizers, biofuels, food supplements, pharmaceutical products, and nutraceuticals, among others. Interestingly, microalgae can synthesize biopolymers, such as polyhydroxybutyrate (PHB) and polyhydroxyalkanoates (PHA) through photosynthetic processes during their cultivation [16, 19]. The relevance of PHB and PHA is based on their likeness to propylene, a highly used oil-based plastic, and in the fact that both biopolymers present a high natural degradation rate in the environment [20, 21].

Despite the remarkable benefits of bioplastic production using microalgae, there are still some current challenges to overcome, such as control of CO_2_, nutrient concentration, and adequate light parameters (e.g., photoperiod, light intensity, type of light); all of them critical factors for large-scale feasible production [22]. Therefore, there is an intense search for new ways to enhance the production of bioresources from microalgae. Some strategies include nutrient control of nitrogen and phosphorus to enhance desired fatty lipids content production [23], variations in light intensity [24], and temperature variations [25]. The regulation of nitrogen and phosphorus nutrients represents a viable avenue for the synthesis of bioplastics by microalgae. This process induces a stress response, causing the accumulation of desired biopolymer constituents. However, this increased accumulation is usually accompanied by a reduction in growth rate, consequently affecting bioplastic production and hindering the scalability of the process [26].

In this context, an innovative nano-technological strategy has been proposed by applying carbon dots (CDs) on the microalgae culture. Recent studies have demonstrated significant improvements with the addition of these carbon-based nanoparticles. For instance, Yang et al. (2022) used graphene oxide quantum dots to increase the photosynthetic activity of *Chlorella pyrenoidosa*; the authors reported an improvement of 20% and 34% in carbon fixation and lipid accumulation, respectively [27]. CDs can be synthesized using various precursors through different methods however, eco-friendly and inexpensive processes are currently required [28]. Accordingly, greener precursors have been explored, such as *Aloe vera* [29], orange juice [30], and prickly pear [31]. Interestingly, the properties and performance of CDs strongly depend on the synthesis methods and the nature of the precursors [32]. Furthermore, CDs’ properties can be further enhanced to improve their performance in different applications; some strategies include element doping and surface passivation [33, 34]. For instance, nitrogen-doped and phosphorous-doped CDs have garnered attention for their biocompatibility in various fields, including bioimaging [35], drug delivery [36], and biosensing [37]. This effect can be attributed to the surface groups, charge interactions, and water dispersibility [38]. Moreover, it has also been reported that co-doping of nitrogen and phosphorus further enhances the biocompatibility properties of CDs [39, 40].

This study obtained green synthesized CDs using prickly pear as the carbon source with a high glucose content [41]. Different CDs were synthesized in solutions rich in nitrogen (NCDs), phosphorus (PCDs), and a combination of nitrogen–phosphorous (NPCDs), which were characterized by different techniques. Then, CDs were added to *Scenedesmus* sp. microalgae cultures to further quantify biomass and PHA production. The effect of CDs addition and the role of their different dopants in bioplastic production by microalgae were carefully analyzed.

## Materials and methods

### Materials

Prickly pears were obtained from a local market, and L-asparagine and phosphoric acid were purchased from Sigma Aldrich. For the synthesis process and culture media, ultra-pure (Milli-Q) and doubled distilled water were used.

### Carbon dots synthesis

First, 1 kg of prickly pear was blended and freeze-dried to reduce the water content, which was used as the carbon source for the CDs synthesis via a hydrothermal approach. Briefly, freeze-dried prickly pear (FDP) was mixed with different concentrations of L-asparagine and phosphoric acid according to Table 1, which then was placed into a 50 mL Teflon-liner autoclave. The concentration of dopants was determined based on existing literature, specifically when prickly pear was used as the precursor for CDs [42]. The hydrothermal conditions were fixed in the three synthesis processes (NCDs, PCDs, and NPCDs), heating at 180 °C for 7 h in a Yamato DKN6026C oven. Synthesis methods were performed by duplicate.

**Table 1:** Conditions for the hydrothermal synthesis of carbon dots

Carbon dots	L-asparagine	Phosphoric acid (0.1 M)	Freeze dried prickly pear
NCDs	0.1 g	–	12.6 g
PCDs	–	10 mL	12.6 g
NPCDs	0.1 g	10 mL	12.6 g

*NCDs* (nitrogen-doped carbon dots), *PCDs* (phosphorous-doped carbon dots), *NPCDs* (nitrogen and phosphorous co-doped carbon dots)

**Fig. 1:**
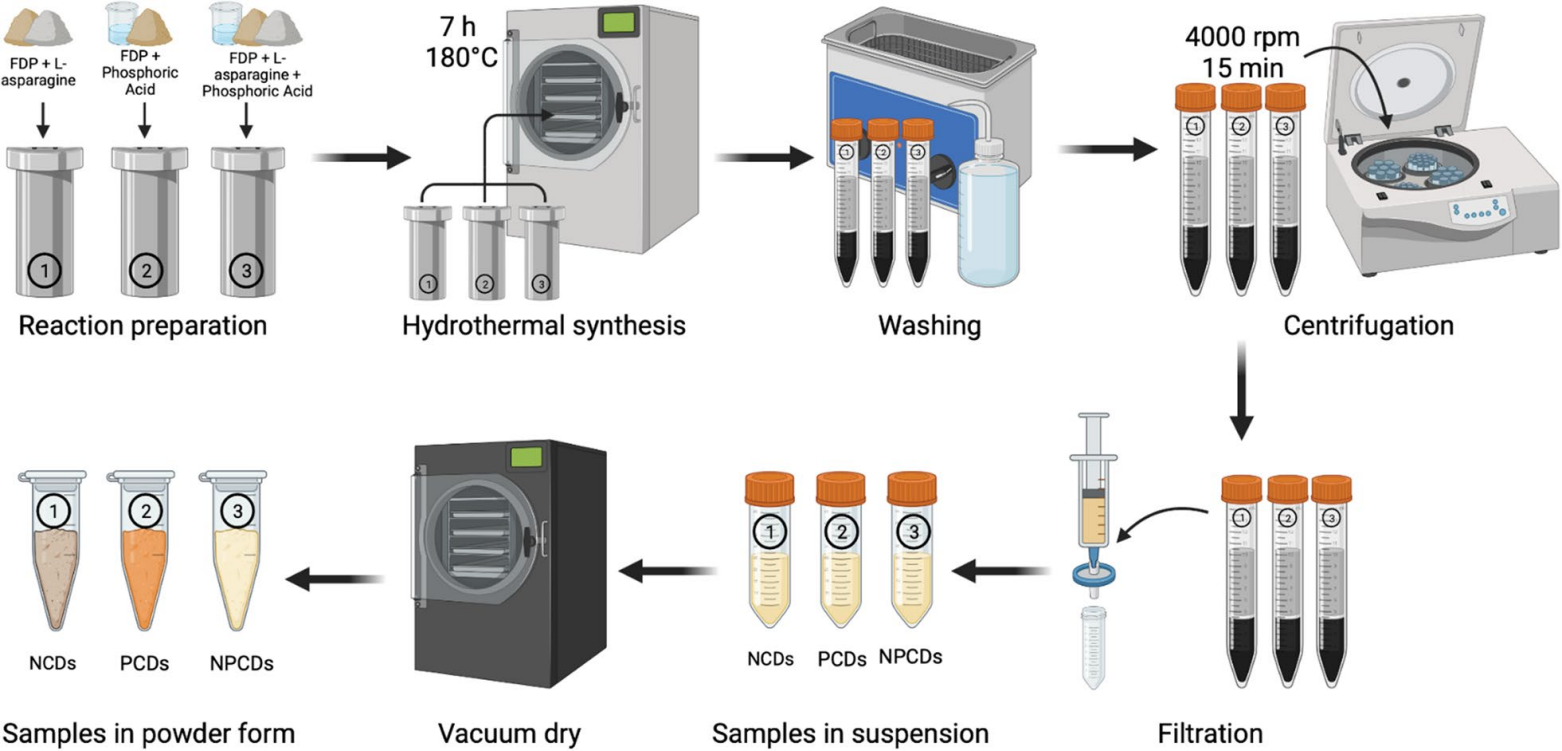
Methodology of the hydrothermal synthesis of carbon dots using prickly pear as the carbon source. *FDP* (Freeze-dried prickly pear), *NCDs* (nitrogen-doped carbon dots, *PCDs* (phosphorous-doped carbon dots, *NPCDs* (nitrogen and phosphorous co-doped carbon dots)

Then, the synthesized brownish and sugary smell-like products were centrifuged four times and washed with Milli-Q water. The suspension was filtered with 0.22 μm filters. Then, samples were vacuum dried on a VO400-memmert oven. All samples were kept in a desiccator for further characterization and analysis. A schematic representation showing the complete methodology is presented in Fig. 1.

### Carbon dots characterization

CDs samples were analyzed by various techniques, i.e., X-ray diffraction (XRD) patterns were analyzed with a Rigaku Miniflex 600 on a current and voltage of 15 mA and 30 kV using Cu K⍺ as the radiation source. Fourier transform infrared (FT-IR) spectroscopy was performed on a PerkinElmer Frontier spectrophotometer. UV–Vis measurements were made with a PerkinElmer UV/Vis Lambda 365 spectrophotometer analyzing between 180 and 700 nm wavelengths. Scanning electronic microscopy (SEM) was performed with a voltage of 20 kV in a Zeiss EVO MA25 and zeta potential with a NanoBrook 90Plus PALS.

### Microalgae culture conditions

*Scenedesmus* sp. strain was purchased from the UTEX Culture Collection of Algae at UT-Austin. Two mediums were used, i.e., (1) BG11 and (2) a modification of BG11 without Nitrogen and Phosphorus. The composition of BG11 as microalgae culture medium was: NaNO_3_ 1.5 g L^−1^, K_2_HPO_4_ 40 mg L^−1^, CaCl_2_ ·2 H_2_O 36 mg L^−1^, MgSO_4_·7 H_2_O 75 mg L^−1^_,_ citric acid 6 mg L^−1^, C_6_H_8_FeNO_7_ 6 mg L^−1^, Na_2_EDTA·2 H_2_O 1 mg L^−1^, Na_2_CO_3_ 20 mg L^−1^, H_3_BO_3_ 2.86 mg L^−1^, MnCl_2_·4 H_2_O 1.81 mg L^−1^, ZnSO_4_·7 H_2_O 0.22 mg L^−1^, Na_2_MoO_4_·2 H_2_O 0.39 mg L^−1^, CuSO_4_·5 H_2_O 0.079 mg L^−1^ and Co(NO_3_)_2_·6 H_2_O 0.04 mg L^−1^.

Batch cultures were prepared with 360 mL of the medium and 40 mL of *Scenedesmus* sp. seeds with a concentration of 1 g/L in 500 mL Erlenmeyer flasks. Different solutions of NCDs, PCDs, and NPCDs were prepared with a concentration of 0.2 mg/L. Then, 2 mL of them were added to the cultures on the first and seventh days. The cultures were filled with double-distilled water on the fifth and tenth days to keep the volume constant of the medium considering the evaporation. For this study, a complete factorial design was performed (Table 2). Continuous atmospheric airflow was maintained in the batch cultures at 21 ± 1 °C, with a 24 h photoperiod using cold white fluorescent lamps. The experiments were performed in triplicate and Table 3 shows the name of the culture with its respective identification.

**Table 2:** The factorial design to determine the effect of CDs’ addition on the cell growth and general characterization of biomass in *Scendesmus* sp. cultures

OrdenEst	Orden	Type of culture	Culture day
2	1	1	2
11	2	4	2
10	3	4	1
3	4	1	3
5	5	2	2
1	6	1	1
7	7	3	1
6	8	2	3
14	9	5	2
8	10	3	2
1	11	5	1
4	12	2	1
15	13	5	3
9	14	3	3
12	15	4	3

Type of culture: BG11 medium (1); BG11 medium without nitrogen and phosphorus (2); BG11 medium without nitrogen and phosphorus with NCDs (3); BG11 medium without nitrogen and phosphorus with PCDs (4); BG11 medium without nitrogen and phosphorus with NPCDs (5). Culture day: 5th day (1); 10th day (2); 15th day (3)

**Table 3:** *Scenedesmus* sp. cultures using different mediums and CDs addition

Culture identification	Medium	CDs
CBG	BG11	–
CBGN-P	BG11 without Nitrogen and Phosphorus	–
CNCDs	BG11 without Nitrogen and Phosphorus	NCDs
CPCDs	BG11 without Nitrogen and Phosphorus	PCDs
CNPCDs	BG11 without Nitrogen and Phosphorus	NPCDs

### Growth measurements of *Scenedesmus* sp. enriched with CDs

The response of the CDs on the microalgae was studied by analyzing the biomass concentration of the samples obtained on the fifth, tenth, and fifteenth day by measuring the optical density of the cultures at 750 nm with a spectrophotometer (Fluorstar omega, 4150470). Different cultures were studied varying the CDs and the medium (Table 3).

### General characterization of microalgae biomass

The *Scenedesmus* sp. batch culture was characterized using 50 mL aliquots of the cultures taken on the fifth, tenth, and fifteenth day, which were stored at 4 °C to be analyzed. Samples were centrifuged at 5000 rpm for 7 min at 10 °C; then, the supernatant was discharged to obtain the biomass. The biomass was washed twice with double-distilled water, and microalgae was allowed to precipitate over a day to avoid biomass loss. Then, it was resuspended in double-distilled water.

Microalgae cultures enriched with CDs were analyzed using the modified phenol–sulfuric method for carbohydrates analysis [43]; proteins were quantified with a Modified Lowry Protein Assay Kit; lipids content was analyzed with sulfo-phospho-vanillin assay [44]. The obtained results of carbohydrate, protein, and lipid contents were statistically analyzed using an analysis of variance (ANOVA) and Tukey’s test by employing Minitab software.

### Total carbohydrate content

The carbohydrate content was determined through the modified phenol–sulfuric method [43]; 300 μL of the resuspended biomass was dissolved in 1 mL of sulfuric acid and subjected to an ice bath for 5 min. The absorbance of the samples was measured at 315 nm using a BMG Labtech microplate reader. The measurements were performed using a glucose standard curve to obtain the carbohydrate content.

### Total protein content

The protein content was measured using the Modified Lowry Protein Assay Kit [45]. The procedure involved adding 1 mL of Lowry’s reagent to 200 μL of biomass, which was then vortexed thoroughly. After 10 min, the procedure was repeated by adding 100 μL of Folin reagent and allowing the reaction for 30 min in a dark area. The absorbance measurements were performed at 750 nm using a BMG Labtech microplate reader and a BSA calibration curve.

### Total lipid content

The lipid content was measured using the sulfo-phospho-vanillin assay [44]. The methodology involved adding 2 mL of sulfuric acid to 100 μL of biomass and heating at 100 °C for 10 min. Then, it was cooled using an ice bath for 5 min. The solution was incubated for 15 min at 37 °C after adding 5 mL of phospho-vanillin reagent. The absorbance measurements were performed at 530 nm using a BMG Labtech microplate reader and a canola oil calibration.

### PHA extraction

The following method is described by [46], which employs the chloroform extraction method. Briefly, dry microalgae biomass was washed with 5 mL and placed in an ultrasonic bath for 30 min. Then, the supernatant was discharged, and 10 mL of hypochlorite solution (4%) was added to the biomass pellet. After incubating it for 1 h at 37 °C, the biomass was centrifuged (8000 rpm, 15 min, 10 °C), and the supernatant was discharged. The biomass was washed twice with double-distilled water under the same conditions. The obtained biomass was transferred to a glass vial and dissolved in 10 mL of boiling chloroform. The chloroform–biomass solution was placed on a condenser for 2 h at 37 °C with a pressure of 15 (pressure unit). Finally, the dry mass was weighed to determine the PHA production. The obtained PHA was analyzed to determine the presence of polyhydroxyalkanoates with an FT-IR spectrophotometer (PerkinElmer Frontier spectrophotometer).

## Results and discussion

### Carbon dots characterization

**Fig. 2:**
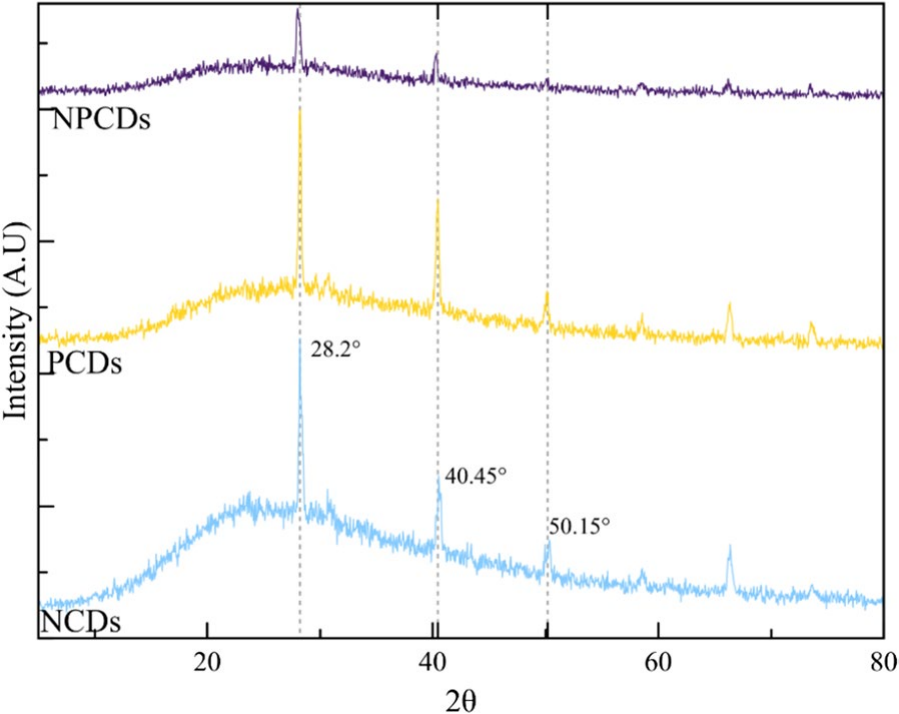
XRD spectra of green prickly pear-derived carbon dots doped with nitrogen (NCDs), phosphorus (PCDs), and nitrogen–phosphorus (NPCDs) co-doped carbon dots showing well-defined peaks at 28.2°, 40.5° and 50.15

Nitrogen, phosphorus, and nitrogen–phosphorus syntheses were proposed to obtain CDs with different dopants and to evaluate their effect on the microalgae PHA production. The synthesized CDs presented a semi-crystalline structure according to the XRD results shown in Fig. 2. Semi-crystalline CDs are reported in the literature, showing better quantum yield values and enhanced photoluminescence properties [47]. It was observed with a characteristic gap of the amorphous structures and well-defined peaks at 28.2°, 40.5°, and 50.15°. NPCDs have the least defined gap among all three samples, suggesting a potential amorphous structure.

The variation in peak intensity and the size of the amorphous gap may be attributed to differences in dopant concentration. The nucleation process of this synthesis remains undescribed; however, other studies on CDs synthesis through pyrolysis and hydrothermal methods have emphasized the significance of pH levels in influencing the nucleation process and the resulting structural characteristics [48–50]. According to the literature, CDs synthesized from glucose can display both amorphous and crystalline structures. For instance, Papaioannou et al. (2018) synthesized glucose-derived CDs via hydrothermal, resulting in crystalline CDs nucleus covered in an amorphous carbon matrix [51]. In addition, a similar XRD pattern is reported by Xu et al. (2021), who observed a potassium–nitrogen–phosphorus hollow structure [52]. Their results are comparable to those obtained in our study, because the prickly pear used as the precursor for CDs contains potassium. In addition, the reactions were conducted by adding nitrogen and phosphorus sources.

**Fig. 3:**
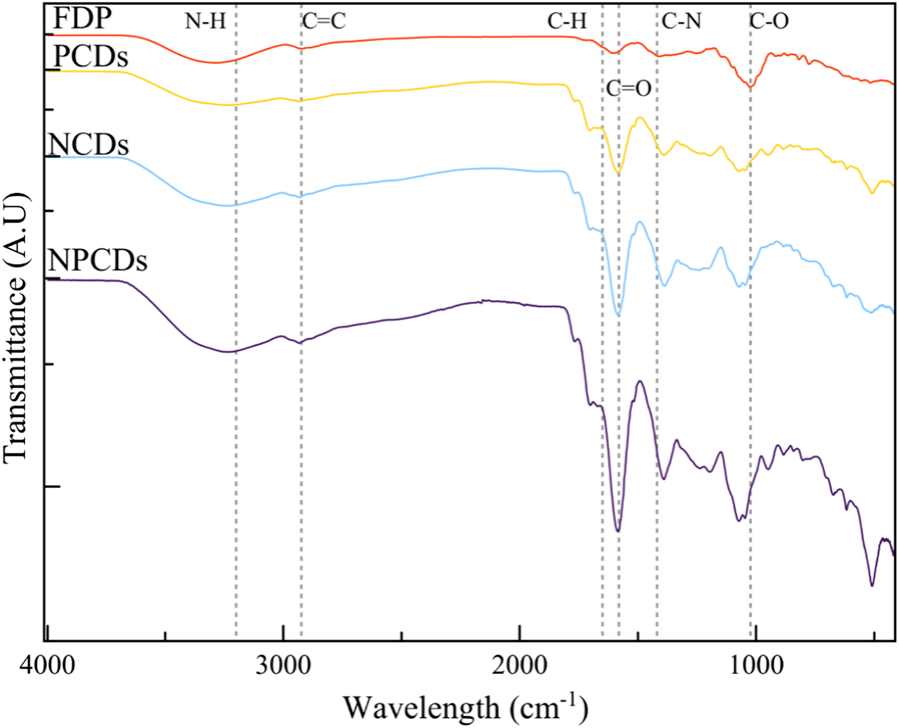
FTIR spectra of freeze-dried prickly pear (FDP) and their derived carbon dots doped with nitrogen (NCDs), phosphorus (PCDs), and nitrogen–phosphorus (NPCDs)

It has been reported that prickly pear is rich in amino acids, sugars, and other components, such as minerals [41]. This complex composition was observed in the FTIR spectra of FDP (Fig. 3). Multiple peaks were detected at 3200 cm^–1^, 1650 cm^–1^, 1580 cm^–1^, 2925 cm^–1^, 1420 cm^–1^, and 1024 cm^–1^, which were attributed to N–H, C–H, C = O, C = C, C–N, C–O, respectively. In addition, it was observed that these functional groups remain on the CDs’ surface; thus, it is suggested a successful addition of carboxyl groups and nitrogen-containing groups to the surface of CDs due to the precursor composition.

**Fig. 4:**
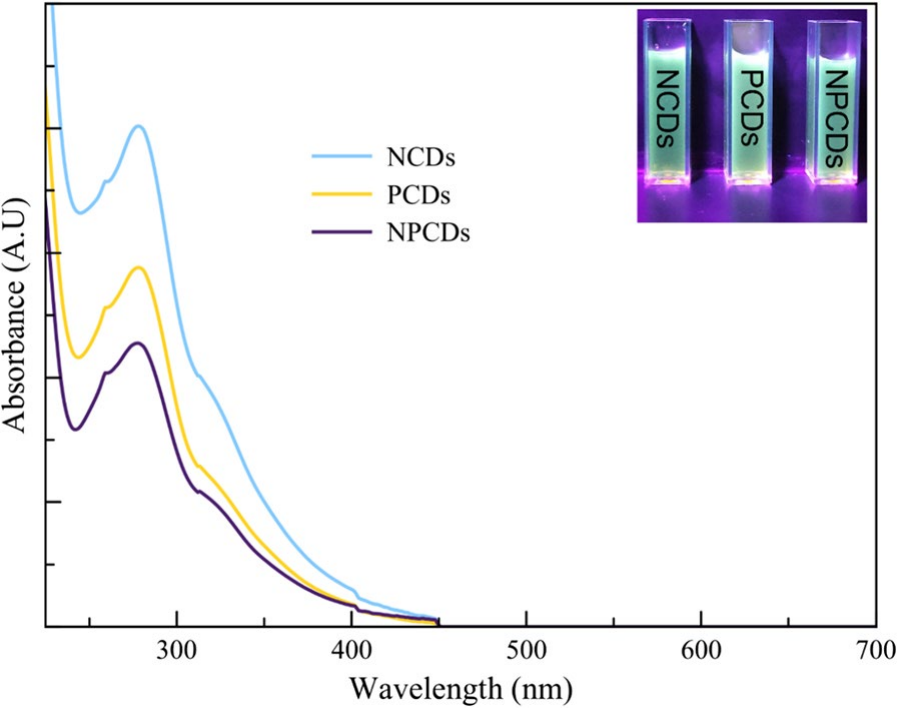
UV–visible absorption spectra of green synthesized prickly pear carbon dots in solutions rich in nitrogen (NCDs), phosphorus (PCDs), and nitrogen–phosphorus (NPCDs)

The FTIR spectra of CDs’ samples show broad bands at 1604 cm^–1^, 1510 cm^–1^, and 1395 cm^–1^, which is typical of five-membered heteroaromatic rings [31] and explains the strong aroma of the prickly pear-derived CDs. There were also peaks related to elements commonly found in the precursor, such as copper; for example, peaks at 940 cm^–1^ and 518 cm^–1^, were detected and attributed to the N–Cu–N band and Cu–O stretching, respectively [47, 53]. Furthermore, NPCDs and PCDs samples presented a band at 880 cm^–1^ and 1370 cm^–1^ attributed to the stretching vibrations of P–O and P = O, suggesting the successful incorporation of phosphorous into the surface of CDs [54, 55]; such bands were not presented in the NCDs sample. Nitrogen element has been widely studied for the functionalization of CDs from multiple precursors and synthesis techniques; nitrogen has been associated with enhanced optical properties. Similarly, phosphoric acid has been reported to strengthen surface functionalization due to increased surface roughness, oxygen-containing polar groups, and the hydrolysis of amino groups [56]. Moreover, phosphoric acid can create new acid surface groups that improve the functionalization of carbon surfaces [57]. Thus, it is suggested that the employment of phosphoric acid can increase the attachment of nitrogen-containing groups when both sources are added during the synthesis. Furthermore, literature reports indicate that variations in nitrogen and phosphorus content during synthesis lead to differences in the FTIR spectra [58–60]. This correlates with the distinct peaks and intensities observed among the samples in the 1000 cm^–^1 region and 500 cm^–1^. Regarding the optical properties, NCDs, PCDs, and NPCDs presented a high absorbance curve in the UV-B and UV-A range, as shown in Fig. 4. The absorption spectra of all CD samples agree with those reported by different authors reporting absorbance in the UV region from UV-C to UV-A. [61]. Furthermore, the UV–Vis absorption spectrum exhibits a prominent absorption peak at 275 nm associated with the orbital $\pi \to {\pi }^{*}$ transition in the carbon core [62].

**Fig. 5:**
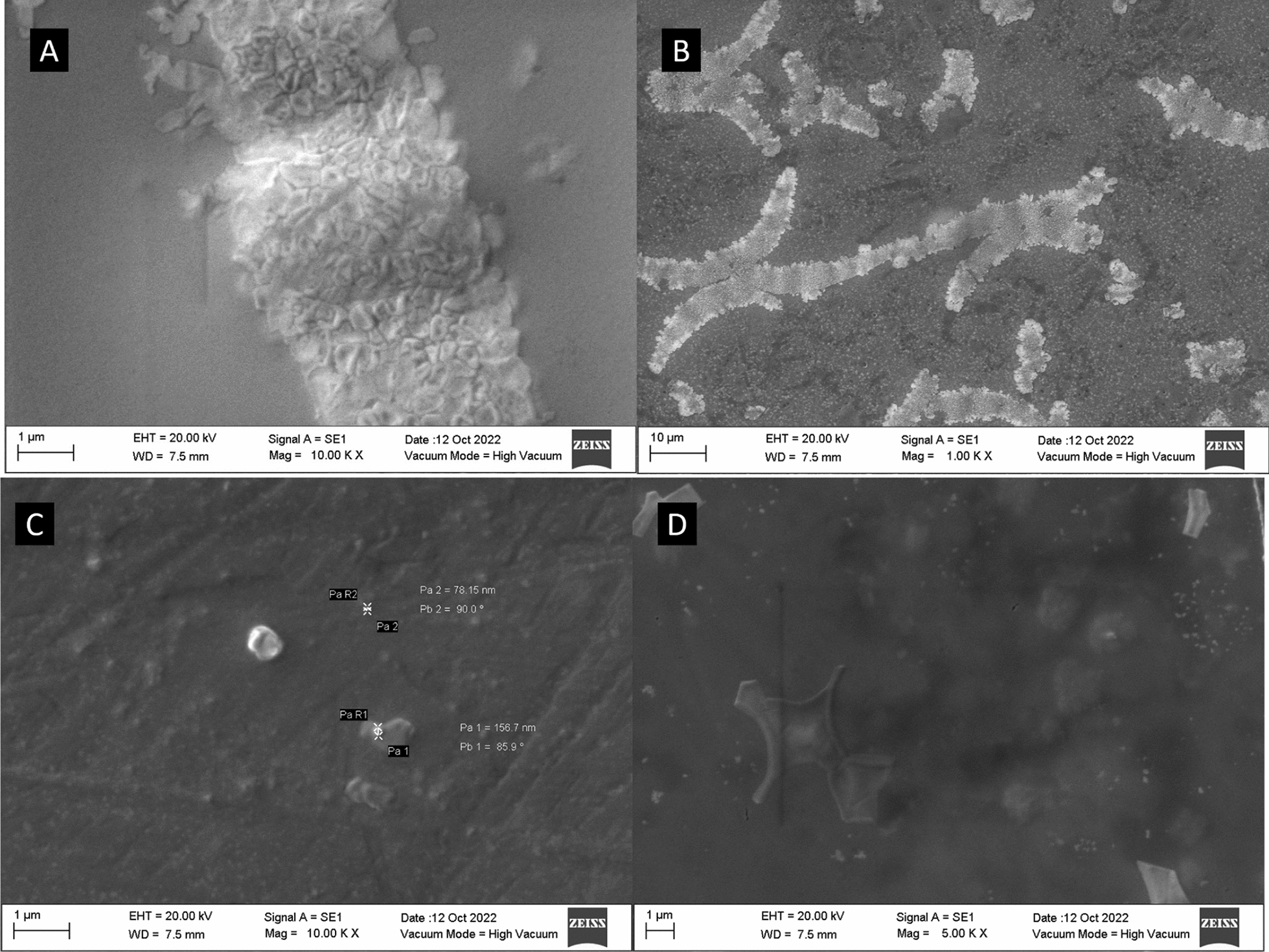
SEM images of green synthesized prickly pear carbon dots. Nitrogen–phosphorous co-doped carbon dots (NPCDs) at (**A**) 1 μm and (**B**) 10 μm. **C** Phosphorous-doped carbon dots synthesized at 1 μm. **D** Nitrogen-doped carbon dots at 1 μm

In terms of morphology, noticeable differences were observed in the SEM images of the CDs samples (Fig. 5). The NPCDs presented a more complex crystal-like structure. In contrast, the morphology of NCDs is better described as semi-spherical agglomerates and PCDs as a mixture of circular agglomerates and crystal-like structures. These observations agree with the results obtained by the XRD technique, since CDs presented a semi-crystalline structure instead of a fully crystalline or amorphous morphology. It was demonstrated that the FTIR and XRD results were similar in crystal planes and composition, but the morphology was completely different. Thus, it is suggested that the various concentration of nitrogen and phosphorus during the synthesis process produces significant differences in the CDs’ morphology.

To analyze the surface charge and the stability of the synthesized prickly pear-derived CDs, it was performed ζ potential measurements. The results presented in Table 4 show poor stability of the CDs samples in the media, mainly in NCDs and PCDs samples; absolute values in the range of 30 or higher are typically reported with highly stable CDs in the media [63]. In this manner and according to ζ potential results, CDs were not showing good stability compared to other reports. However, dry CDs samples showed a highly high hydrophilic behavior during the characterization stage; samples in suspension did not show precipitation after long periods. All CDs samples presented a negative charge, indicating a dense electron cloud associated with the presence of functional groups, such as carbonyl, hydroxyl, and carboxyl in the CDs’ surface [64], which agrees with FTIR results.

**Table 4:** ζ potential results of prickly pear-derived carbon dots

Sample	ζ potential (mV)		
Nitrogen-doped carbon dots	− 0.41	±	0.77
Phosphorus-doped carbon dots	− 6.69	±	2.76
Nitrogen–phosphorus multi-doped carbon dots	− 5.30	±	1.37

### Growth behavior and biomass production of *Scenedesmus* sp. microalgae culture

**Fig. 6:**
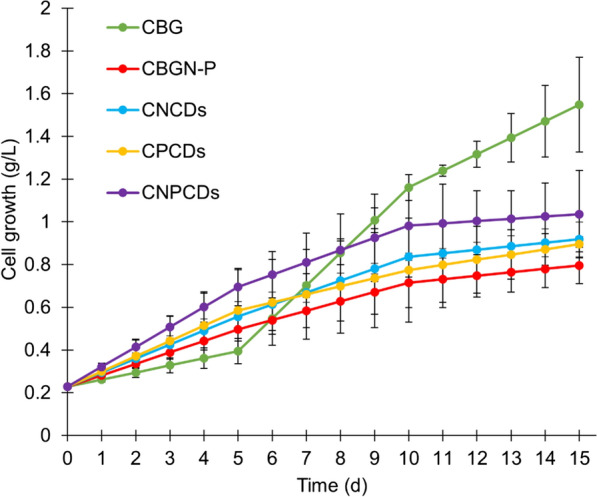
Cell growth of *Scenedesmus* sp. microalgae enriched with green prickly pear-derived carbon dot*:* (CBG), modified BG11 medium (CBGN-P), and cultures enriched with nitrogen-doped carbon dots (CNCDs), phosphorous-doped carbon dots (CPCDs), and nitrogen phosphorous co-doped carbon dots (CNPCDs)

CDs samples were added in microalgae cultures to evaluate the growth behavior of *Scenedesmus* sp. and the effects of CDs (Fig. 6). As expected, the blank culture with BG11 medium (CBG) presented the best growth behavior due to its medium content with no modifications of nitrogen and phosphorus, which are vital elements for the natural growth of microalgae [65]. The control CBG presented the highest cell growth, specifically on the 14th and 15th days. The CBG reached a growth of 1.5486 g L^–1^, 94% more than the BG11 medium without nitrogen and phosphorus (CBGN-P). CBGN-P medium showed the lowest growth rate; however, it has demonstrated great potential to improve PHA production [45]. Therefore, CBGN-P medium was included in this study to compare with the CDs-enriched mediums regarding PHA production.

All the CDs-enriched cultures exhibited better growth behavior than the CBGN-P culture, as reported by [27]. Moreover, the CNPCDs culture presented the best growth behavior among all CDs-enriched and CBGN-P mediums, with 32% higher concentration than CBGN-P. A 32% increase in cellular growth represents a significant improvement in the microalgae culture. In comparison, other cultures of microalgae using bacteria and nutrient control to enhance cellular growth in BG11 medium achieved only a 29% and 18% increase in cellular growth (g/L) [66, 67].

**Fig. 7:**
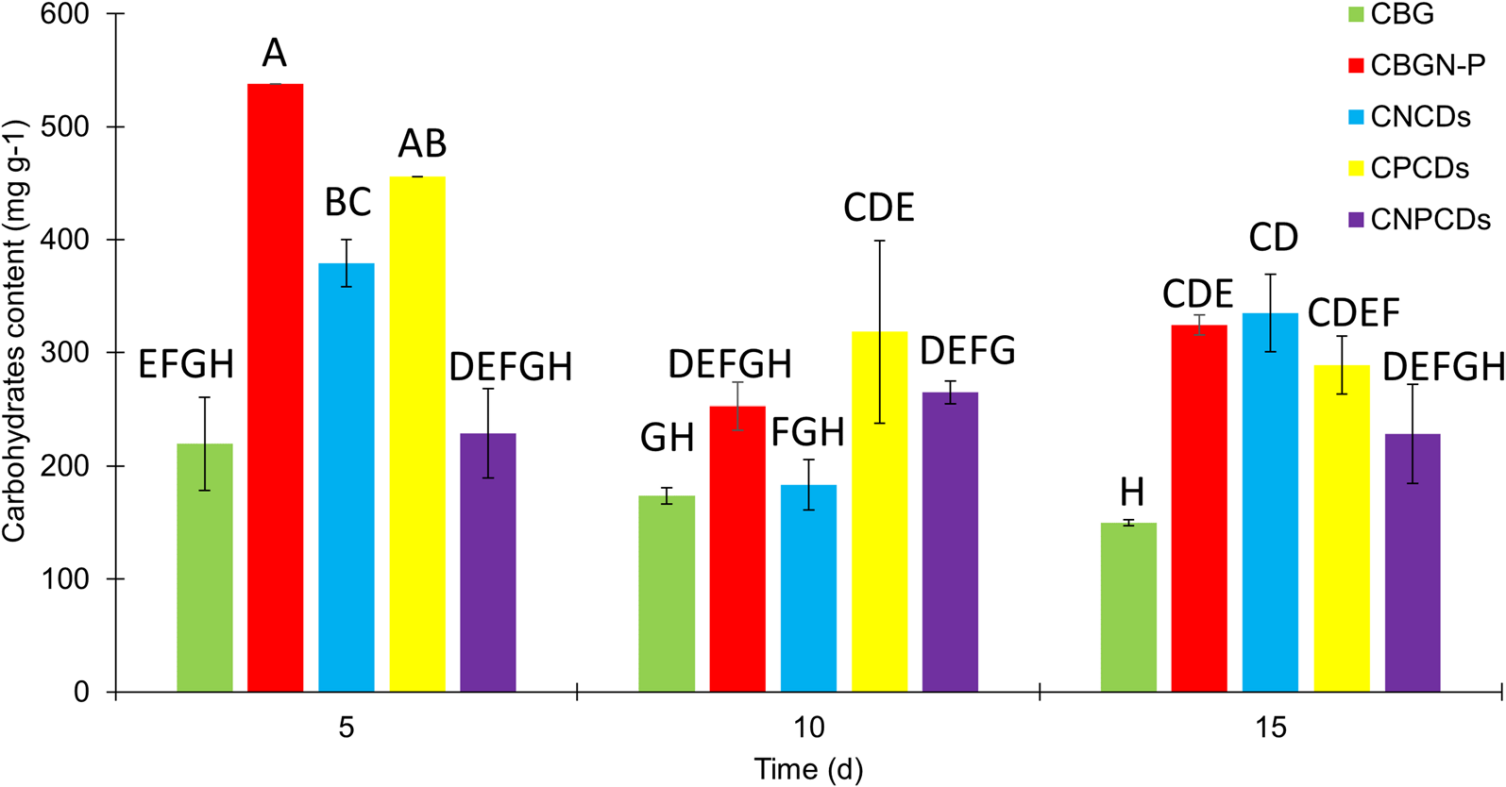
Carbohydrates analysis of *Scenedesmus* sp. cultures in BG11 medium (CBG), modified BG11 medium (CBGN-P), and cultures enriched with nitrogen-doped carbon dots (CNCDs), phosphorous-doped carbon dots (CPCDs), and nitrogen phosphorous co-doped carbon dots (CNPCDs). Note: The statistics were obtained from a multi-factor ANOVA followed by Tukey’s test. Different letters above the bars denote the significant difference at* p* < 0.05

The carbohydrate content analysis presented in Fig. 7 demonstrates that adding CDs—regardless of the doping element—did not represent a significant enhancement in the production. It was reported that 33.49% of the dry weight in the CNCDs was slightly higher than that obtained by CBGN-P (32.44%). Contrarily, CPCDs, and CNPCDs did not present a higher carbohydrate accumulation than CBGN-P; those cultures presented 28.88% and 22.83%, respectively. The first days are crucial to obtaining the most carbohydrate, which can be observed by the decrease after 10 and 15 days of cultivation. On the 5th day, CBGN-P showed the highest carbohydrate content, significantly different from all enriched-CDs mediums, except for CPCDs. On the 10th day, CPCDs showed the highest carbohydrate content, substantially different from CNCDS and CNPFCDs. Finally, on day 15th, CNCDs showed the highest carbohydrate content, statistically different from CPCDs and CNPCDs. The addition of CDs does not lead to a notable increase in carbohydrates compared to other microalgae culture parameters, such as light intensity, temperature, and nitrogen control in BG11 medium [68, 69]. However, the observed carbohydrate levels are within the range reported for microalgae cultures focused on lipid and bioplastic production [70].

**Fig. 8:**
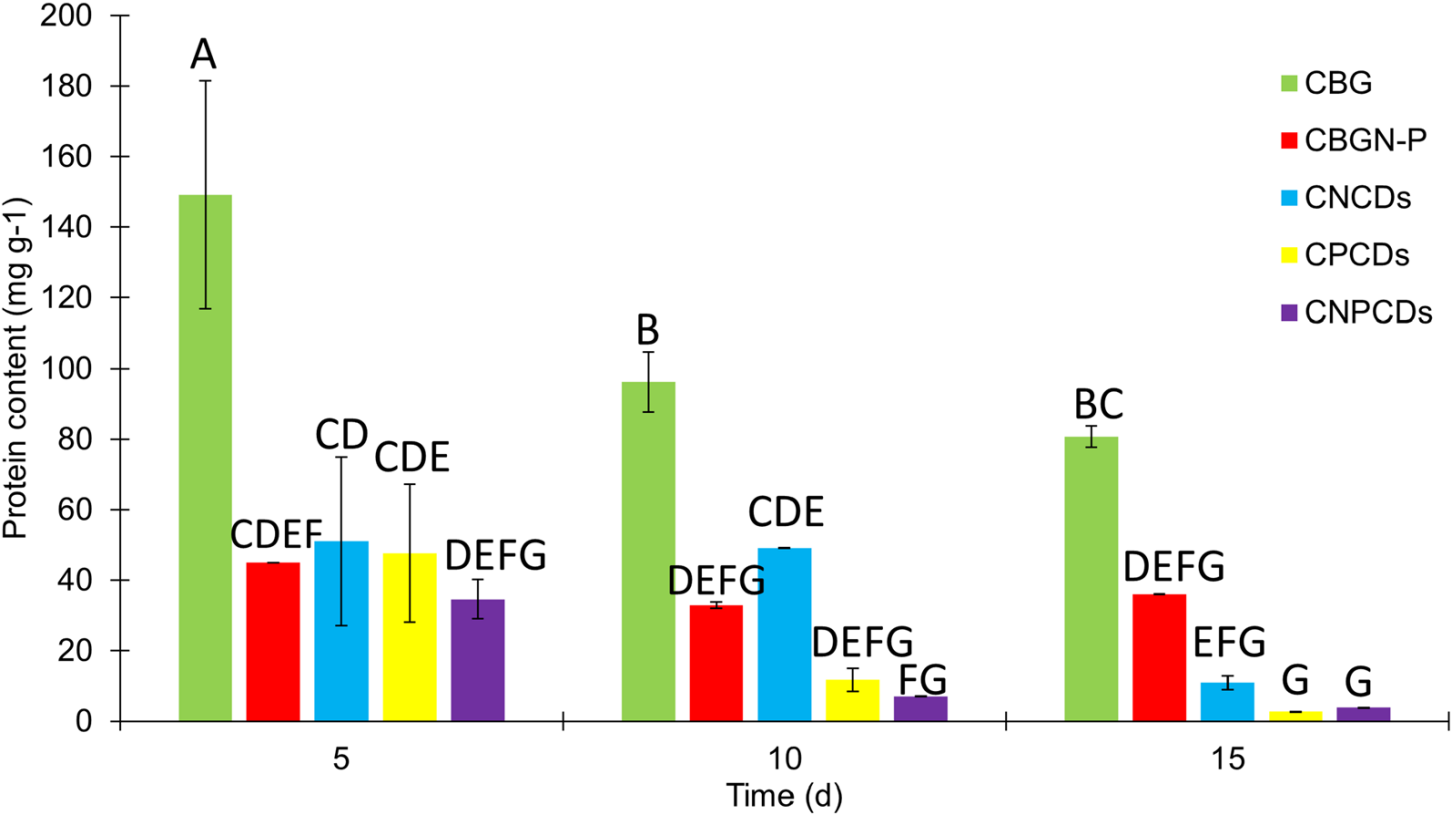
Protein analysis of *Scenedesmus* sp. cultures in BG11 medium (CBG), modified BG11 medium (CBGN-P), and cultures enriched with nitrogen-doped carbon dots (CNCDs), phosphorous-doped carbon dots (CPCDs), and nitrogen phosphorous co-doped carbon dots (CNPCDs). Note: The statistics were obtained from a multi-factor ANOVA followed by Tukey’s test. Different letters above the bars denote the significant difference at* p* < 0.05

On the other hand, the microalgae cultures enriched with CDs showed a significant decrease in the total protein content compared to control cultures (CBG and CBGN-P). As observed in Fig. 8 CPCDs produced almost no protein content, while CNCDs and CNPCDs produced around 30% of the total protein produced by CBGN-P. This result can be explained by the fact that nitrogen compounds usually represent up to 30% of cellular biomass; they perform important functions, including the production of proteins [71]. In this context, the lower protein content in CPCDs can be associated with the fact that nitrogen was not added to those cultures; thus, CPCDs barely reached 0.27% of the dry weight, while CBGN-P presented 3.61% and CBG exhibited the highest value of 8.07%. On the 5th day, CBG showed the highest value, which was significantly different from all the cultures; the CBGN-P control medium showed a lower value; however, it did not show a significant difference between CNCDs and CPCDs. Statistically, CBG did not present differences in the protein content after 10 and 15 days of culture. Moreover, a similar pattern was observed, since the CBG medium showed the highest value, significantly different from all modified cultures (CBGN-P and CDs-enriched mediums). The protein content in cultures enriched with CDs exhibits a remarkable decrease compared to studies reported in the literature [72]. It has been reported a reduction in protein content in the presence of metals, such as copper [73, 74], which aligns with the detection of copper in the CDs as identified by FTIR analysis. Therefore, it is suggested that the presence of copper in the CDs could have impacted the protein content.

**Fig. 9:**
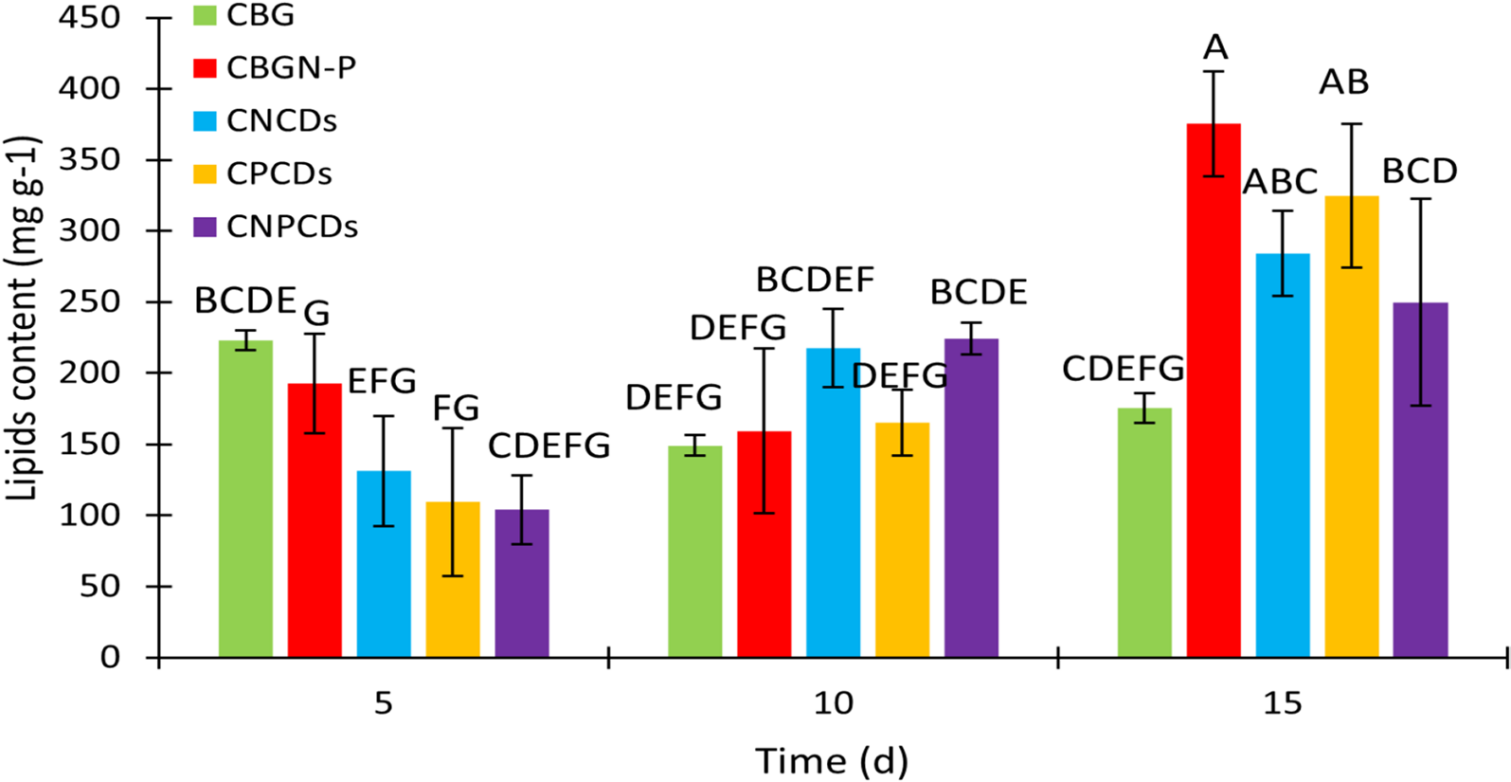
Analysis of the lipid content of *Scenedesmus* sp. cultures in BG11 medium (CBG), modified BG11 medium (CBGN-P), and cultures enriched with nitrogen-doped carbon dots (CNCDs), phosphorous-doped carbon dots (CPCDs), and nitrogen phosphorous co-doped carbon dots (CNPCDs). Note: The statistics were obtained from a multi-factor ANOVA followed by Tukey’s test. Different letters above the bars denote the significant difference at* p* < 0.05

Regarding the lipids content, CBGN-P, CPCDs, and CNCDs showed the highest lipid production after 15 days of cultivation, with no significant differences between them. The percentage values of the dry weight obtained by CBGN-P, CPCDs, and CNCDs were 37.53%, 28.88%, and 28.42%, respectively (Fig. 9). CNCDs and CNPCDs cultures exhibited an approximate reduction of 13% in comparison with that obtained by CBGN-P. Whereas the lipid content of CPCDs was slightly reduced (3%). The higher lipid accumulation in CPCDs compared to CNCDs and CNPCDs is associated with the relevant role played by phosphorus in lipids production [75].

### PHA production by *Scenedesmus* sp. microalgae cultures enriched with carbon dots.

**Fig. 10:**
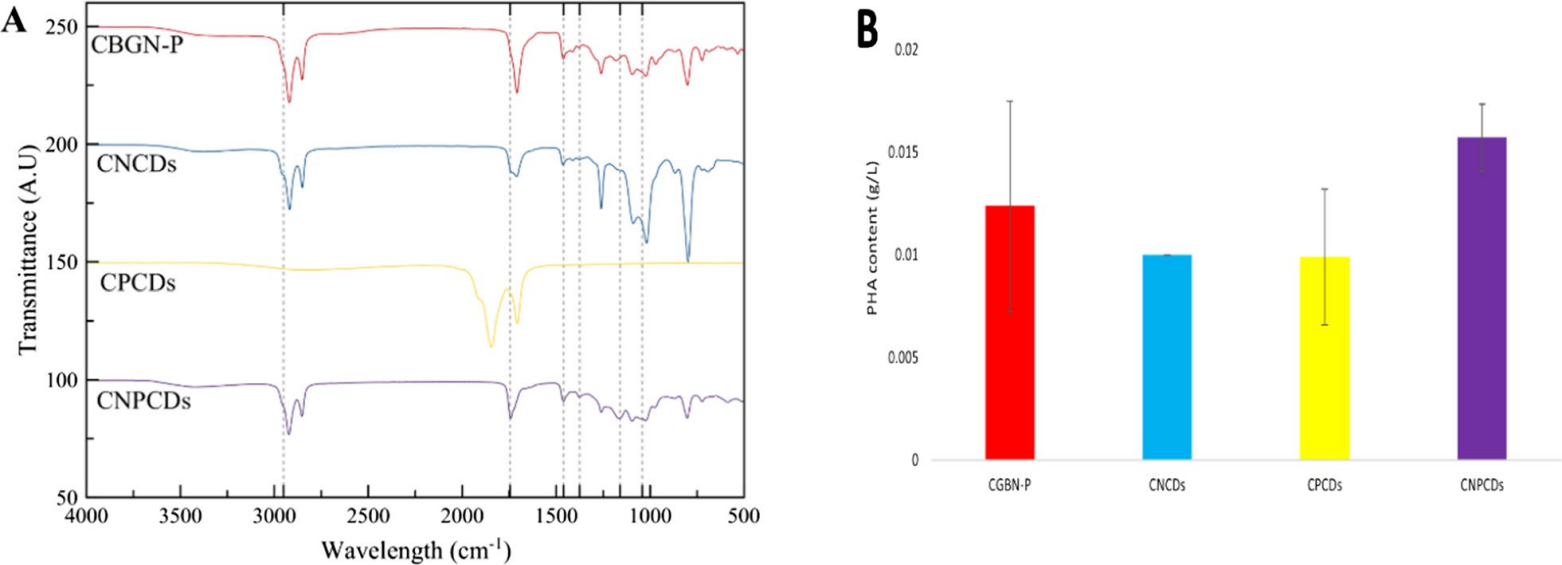
Analysis of the PHA production of *Scenedesmus* sp. cultures. **A** FTIR spectra of the PHA produced by the microalgae cultured in modified BG11 medium (CBGN-P), and cultures enriched with nitrogen-doped carbon dots (CNCDs), phosphorous-doped carbon dots (CPCDs), and nitrogen phosphorous co-doped carbon dots (CNPCDs). **B** Analysis of the PHA production by *Scenedesmus* sp. cultures in modified BG11 medium (CBGN-P), and cultures enriched with nitrogen-doped carbon dots (CNCDs), phosphorous-doped carbon dots (CPCDs), and nitrogen phosphorous co-doped carbon dots (CNPCDs)

The presence of PHA was confirmed with the FTIR spectra of CNCDs, CNPCDs, and CBGN-P (Fig. 10A). All samples showed a strong band at 1746 cm^–1^, ascribed to the vibration band of C–O–C. Other peaks were observed in the CNCDs, CNPCDs, and CBGN-P cultures, such as the ones reported at 1374 cm^–1^, 1460 cm^–1^, and 2952 cm^–1^, which suggest the presence of –CH_3_, –CH_2_, and –CH groups, respectively. Interestingly, CNCDs presented a high vibration band between 1023 cm^–1^ and 1040 cm^–1^, which is related to the C–O stretching and PHB formation, which can be expected according to reports from other authors [45].

Regarding PHA quantification, CNPCDs exhibited the highest PHA accumulation (Fig. 10B). Nevertheless, it presented a lower percentage of PHA per gram of microalgae compared to CBGN-P with 6% of dry weight, which is in agreement with other reports [45]. CNCDs, CPCDs, and CNPCDs presented a PHA percentage from dry weight of 4.1%, 4.7%, and 5.7%, respectively. Interestingly, the highest concentration of PHA was obtained by the *Scenedesmus* sp. culture enriched with NPCDs (CNPCDs culture), which produced 26.92% more PHA than control CBGN-P, which has demonstrated the potential to increase PHA production in other reports [27]. This result can be associated with the cellular growth surpassing the control conditions, compensating for the PHA presented per gram with a higher concentration of microalgae. Further studies should consider variations in CDs’ dosages to evaluate the concentration effect on PHA production. In addition, more research should be directed to understand the precise mechanisms and the role played by the CDs, since they can act both as a nutrient source for microalgae growth or as an additive with excellent optical properties to enhance the photosynthetic capabilities of microalgae.

## Conclusions

This work demonstrates the successful green hydrothermal synthesis of CDs doped with multiple elements using pricky pear as the carbon precursor. Prickly pear is an excellent alternative to produce CDs owing to its rich glucose and mineral content, inherent self-functionalization, and enhanced optical properties. In this study, prickly pear-derived CDs were doped with nitrogen (NCDs), phosphorous (PCDs), and a combination of nitrogen–phosphorous (NPCDs). CDs were synthesized for their further addition into *Scenedesmus* sp. microalgae culture.

The addition of the synthesized CDs led to significant differences; for example, NCDs caused higher carbohydrate accumulation, while PCDs increased the lipid content. Moreover, NPCDs enrichment resulted in the highest PHA concentration, exhibiting a remarkable 26.92% increase compared to the modified-control BG-11. These findings emphasize the potential of CDs to modulate the accumulation of molecules in microalgae cultures, with even small quantities yielding substantial differences compared to conventional cultures.

Therefore, CDs-enriched cultures are proposed as an excellent alternative for obtaining valuable products. However, future research should explore additional parameters such as CDs concentration, their toxicity in microalgae, interaction with nutrients, and uptake by microalgae. This study paves the foundation for further investigations that could provide a viable solution for large-scale bioplastic production without additional equipment or large quantities of materials.

## Data Availability

Data availability statement is not applicable.

## Abbreviations

CBG: BG11 Medium
CBGN-P: BG11 without Nitrogen and Phosphorus
CDs: Carbon dots
CNCDs: BG11 without Nitrogen and Phosphorus and NCDs
CNPCDs: BG11 without Nitrogen and Phosphorus and NPCDs
CPCDs: BG11 without Nitrogen and Phosphorus and NPDs
FDP: Freeze-dried prickly pear
FT-IR: Fourier transform infrared
NCDs: Nitrogen-doped carbon dots
NPCDs: Nitrogen and phosphorous co-doped carbon dots
PCDs: Phosphorous-doped carbon dots
PHA: Polyhydroxyalkanoates
PHB: Polyhydroxybutyrate
SEM: Scanning Electronic Microscopy
XRD: X-ray diffraction
